# Cholesterol and HIF-1α: Dangerous Liaisons in Atherosclerosis

**DOI:** 10.3389/fimmu.2022.868958

**Published:** 2022-03-21

**Authors:** Charles Thomas, Damien Leleu, David Masson

**Affiliations:** ^1^Univ. Bourgogne Franche-Comté, LNC UMR1231, Dijon, France; ^2^INSERM, LNC UMR1231, Dijon, France; ^3^LipSTIC LabEx, Dijon, France; ^4^CHRU Dijon Bourgogne, Laboratory of Clinical Chemistry, Dijon, France

**Keywords:** cholesterol, atherosclerosis, macrophage, oxysterol, hypoxia inducible factor (HIF), liver X receptor

## Abstract

HIF-1α exerts both detrimental and beneficial actions in atherosclerosis. While there is evidence that HIF-1α could be pro-atherogenic within the atheromatous plaque, experimental models of atherosclerosis suggest a more complex role that depends on the cell type expressing HIF-1α. In atheroma plaques, HIF-1α is stabilized by local hypoxic conditions and by the lipid microenvironment. Macrophage exposure to oxidized LDLs (oxLDLs) or to necrotic plaque debris enriched with oxysterols induces HIF-1α -dependent pathways. Moreover, HIF-1α is involved in many oxLDL-induced effects in macrophages including inflammatory response, angiogenesis and metabolic reprogramming. OxLDLs activate toll-like receptor signaling pathways to promote HIF-1α stabilization. OxLDLs and oxysterols also induce NADPH oxidases and reactive oxygen species production, which subsequently leads to HIF-1α stabilization. Finally, recent investigations revealed that the activation of liver X receptor, an oxysterol nuclear receptor, results in an increase in HIF-1α transcriptional activity. Reciprocally, HIF-1α signaling promotes triglycerides and cholesterol accumulation in macrophages. Hypoxia and HIF-1α increase the uptake of oxLDLs, promote cholesterol and triglyceride synthesis and decrease cholesterol efflux. In conclusion, the impact of HIF-1α on cholesterol homeostasis within macrophages and the feedback activation of the inflammatory response by oxysterols *via* HIF-1α could play a deleterious role in atherosclerosis. In this context, studies aimed at understanding the specific mechanisms leading to HIF-1α activation within the plaque represents a promising field for research investigations and a path toward development of novel therapies.

## Introduction

The hypoxia inducible factor (HIF)-1α transcription factor is a major regulator of the cellular response to hypoxia. As a heterodimer with HIF-1β it contributes to the adaptation to hypoxic conditions by binding to key target gene promoters such a vascular endothelial growth factor (VEGF) and erythropoietin (1, 2). Under normoxic conditions, HIF-1α is rapidly hydroxylated at specific proline residues and degraded by the proteasome. Under hypoxic conditions, the activity of prolyl-hydroxylase domain (PHD) enzymes is inhibited which leads to the stabilization of HIF-1α and subsequent translocation to the nucleus (3, 4).

While cholesterol accumulation in myeloid cells is a major driver of the atherogenic process, hypoxia and activation of HIFs are now recognized as important players (5–9). Interestingly, HIF-1α has been shown to impact lipid homeostasis in macrophages and to promote cholesterol accumulation (10). Reciprocally, cholesterol-derived molecules such as oxysterols, present in the atheroma plaque or originating from oxidized low density lipoproteins (oxLDLs) can trigger HIF-1α activation in macrophages with consequences on interleukin-1β (IL1-β) production and the metabolic rewiring of macrophages with an induction of glycolysis and glucose uptake (11–13).

We review here some studies that investigated the regulation of cholesterol metabolism by hypoxia and HIF-1α as well as the mirror regulation of HIF-1α signaling by cholesterol and related derivatives. Finally, we discuss the consequences of theses “dangerous liaisons” in the pathogenesis of atherosclerosis and the potential interest of pharmacological targeting the HIF-1α-cholesterol axis.

## HIF-1α and Atherosclerosis

The relationship between HIF-1α and atherosclerosis is well documented and has been reviewed previously (14, 15). While several levels of evidence support the hypothesis that HIF-1α could be pro-atherogenic within atheroma plaques (10), experimental models of atherosclerosis in mice suggest a complex role that depends on the cell type or the organ in which HIF-1α is present (16, 17).

HIF-1α is expressed and functional in most of the cells that contribute to plaque formation and progression: endothelial cells, vascular smooth muscle cells (VSMCs) and immune cells such as macrophages (16, 18, 19). In human atherosclerotic plaques, immuno-histology experiments show that HIF-1α is mainly detected in macrophages, in pro-inflammatory regions rich in lipids and potentially hypoxic (20). Expression of HIF-1α in human plaques is associated with pro-angiogenic and pro-inflammatory factors, VEGF and IL-1β, respectively (12, 20, 21). HIF-1α expression also correlates with two markers of plaque instability: intra-plaque hemorrhages and plaque angiogenesis (21–23). Recently, single cell RNA sequencing studies in mice further confirmed that HIF-1α is expressed in different macrophage subsets as well as in monocytes within the plaque (24, 25).

Experimental studies suggest that HIF-1α activation in different cell types present within the plaque is mainly associated to pro-atherogenic effects. In murine endothelial cells, activation of HIF-1α increases the expression of VEGF and its receptors as well as the production of nitric oxide (NO) through NO synthase induction (26, 27). Hypoxia is also associated with the production of reactive oxygen species (ROS) by endothelial cells *via* the activation of the HIF-1α target genes NADPH oxidases (NOXs) (28–30). Overall, HIF-1α-dependent pathways induce endothelial cell dysfunction and increase endothelial inflammation, which could subsequently promote the adhesion and recruitment of immune cells (16). In VSMCs, hypoxia induces their migration and their proliferation, through the recruitment of HIF-1α which will activate several target genes including migration inhibitory factor, VEGF and thrombospondin-1 (31–33). Because VSMCs functions in atherosclerosis are highly complex (34, 35), the consequences of HIF-1α-induced VSMCs migration and proliferation are yet to be determined.

In the context of atherosclerosis, one of the best-documented effects of HIF-1α is its action on macrophages. Notably, HIF-1α regulates the expression of pro-angiogenic genes such as VEGF (36) and contributes to the metabolic reprogramming of macrophages by activating glucose uptake and glycolysis which are hallmarks of plaque macrophage metabolism (37). HIF-1α induces significant alterations in macrophage lipid metabolism, leading to lipid accumulation (10). HIF-1α signaling plays a major role in the regulation of macrophage activation and inflammatory response and promote macrophage polarization toward an M1 phenotype (38, 39). IL-1β, a pro-inflammatory cytokine, is a direct HIF-1α target (40). In activated macrophage, increased levels of succinate stabilize HIF-1α and allow the induction of IL-1β (41). The HIF-1α-IL-1β axis is of peculiar interest in the context of atherosclerosis. Indeed IL-1β is a promising therapeutic target and strategies aimed at inhibiting IL-1β with monoclonal antibodies have led to a significantly lower rate of cardiovascular events in high-risk patients (42).

Mouse models of atherosclerosis can be used to provide a more direct assessment of the contribution of HIF-1α to atherogenesis. Interestingly, the impact seems to vary depending on the cell type considered. Low density lipoprotein receptor-deficient mice (*Ldlr^-/-^*) mice transplanted with the bone marrow of mice deficient for *Hif1a* in myeloid cells had a 72% reduction in atheromatous lesions in the aorta. Conversely, bone marrow transplantation from mice presenting a constitutive activation of HIF-1α (mice deficient for Von Hippel–Lindau tumor suppressor) increased atherosclerosis (18). Another study in *Ldlr^-/-^* mice found that cell-specific deletion of *Hif1a* in LysM+ bone marrow cells did not affect the formation of atherosclerotic lesions whereas the conditional invalidation of *Hif1a* in dendritic cells (cd11c+ cells) accelerated atherosclerotic plaque formation and increased T cell infiltration (43). In line with a potentially beneficial role, HIF-1α overexpression in mouse lymphocytes was associated with a reduction of IFN-γ expression and a reduced development of atherosclerosis (17).

Regarding non-immune cells, *Hif1a* deletion in endothelial cells in apolipoprotein E-deficient (*ApoE-/-)* mice led to a decrease in atheromatous lesions and macrophage accumulation in carotids and the aorta (16). Finally, in the same model of atherosclerosis-sensitive mice, specific *Hif1a* deletion in smooth muscle cells reduced vascular inflammation and atherosclerosis (19).

While these studies show contrasting effects of HIF-1α depending on cell type, *Ldlr-/-* mice treated with an inhibitor of HIF prolyl 4-hydroxylase-2 leading to the stabilization of HIF-1α and HIF-2α were found to have decreased plasma lipid levels and attenuated atherosclerosis development (44). These data suggest that HIF-1α exerts systemic beneficial effects in the liver and the adipose tissue with favorable consequences on atherosclerosis development (44).

## Regulation of HIF-1α by Cholesterol and Lipid Microenvironment

### Mechanisms of HIF-1α Activation Within the Atheromatous Plaque

Local hypoxic conditions within specific plaque areas certainly play a major role in HIF-1α activation (6, 20, 37, 45). Nevertheless, it appears that other factors associated with the macrophage microenvironment, also contribute significantly to the activation of HIF-1α. Pro-inflammatory cytokines and chemokines can be detected within the plaque, as well as high levels of cholesterol and oxidized lipids (oxysterols, lysophospholipids and oxidized phospholipids). A variety of damage associated molecular patterns released by dying cells are also present (46). Therefore, inflammation and toll-like receptor (TLR) dependent pathways can also induce HIF-1α signaling by several complementary mechanisms. HIF-1α is a NF-κB target gene, therefore TLR signaling activates HIF-1α at the transcriptional level. ROS production secondary to TLR activation also promotes HIF-1α stabilization (47–49). Finally, the metabolic reprogramming of inflammatory macrophages leading to the accumulation of succinate also activates HIF-1α (41).

**Figure 1:**
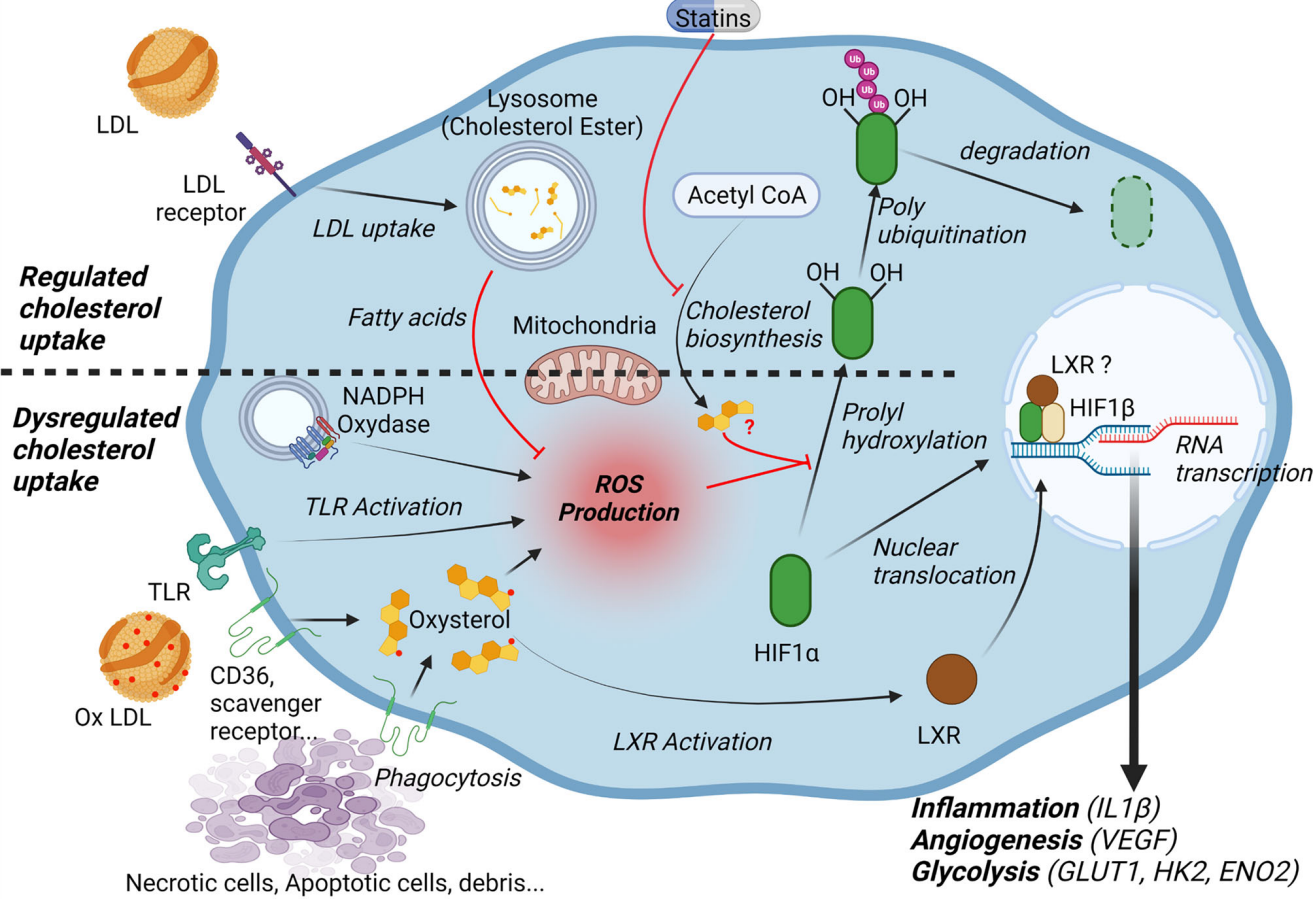
Cholesterol and lipid microenvironment regulate HIF-1α. Low density lipoproteins (LDL) are taken up into the cells *via* endocytosis of the LDL receptor (LDLR). Fatty acids released by the lysosomal hydrolysis of cholesteryl esters inhibit mitochondrial reactive oxygen species (ROS) production and decrease HIF1α stability. Cholesterol synthesis may also affect HIF-1α dependent pathways through the generation of isoprenoids and subsequent isoprenylation of G-proteins. In the context of atheroma, macrophages engulf oxidized lipoproteins and oxysterol-rich cellular debris, which leads to ROS production and increases HIF-1α stability. Moreover, oxysterol accumulation activates the Liver X Receptor (LXR). LXR may directly interact with HIF-1α. Overall, HIF-1α activation promotes the expression of genes implicated in inflammation (interleukin 1-β), angiogenesis (vascular endothelial growth factor (VEGF) and glycolysis (glucose transporter 1 (GLUT1), hexokinase 2 (HK2), enolase 2 (ENO2).

Recent studies also demonstrate that the lipid microenvironment, in particular the high concentrations of cholesterol and its oxidized derivatives plays a significant role in the activation of HIF-1α signaling. Accumulation of oxLDLs in the intima of arteries and their uptake by macrophages is a hallmark of atherosclerosis (50, 51). Macrophages engulf oxidized lipoproteins through different scavenger receptors without feedback inhibition by cholesterol (50, 51). Alternatively, macrophages can also acquire cholesterol and oxidized derivatives *via* efferocytosis and phagocytosis of cholesterol-rich cellular debris (12). In the context of the atheromatous plaque, native LDL uptake or endogenous cholesterol biosynthesis, two retro-regulated processes, certainly play less important roles (50, 51). Interestingly, various studies have shown that these different cholesterol supply pathways are likely to modulate the activation of HIF-1α (13, 52). Nevertheless, as discuss below, their impact differs greatly if one considers the classic retro-regulated pathways *i.e.* cholesterol biosynthesis or uptake of native LDL (52, 53), or the pathways that are likely to be favored within the plaque, *i.e.* oxLDL uptake or phagocytosis of cellular debris (**Figure 1**) (12, 13).

### Regulation of HIF-1α by Cholesterol Biosynthesis and Lipoprotein Uptake

Various studies have focused on the impact of statins, HMGCoA reductase inhibitors and cholesterol-lowering agents on HIF-1α (53, 54). In VSMCs, fluvastatin was found to inhibit the expression HIF-1α target genes under hypoxic conditions by accelerating HIF-1α ubiquitination (53). This could be explained by a decrease in isoprenoids (mevalonate, farnesyl-pyrophosphate) and in the prenylation of several G-proteins in statin-treated cells (53). By contrast, in endothelial cells, simvastatin increases both HIF-1α and VEGF expression. Mechanistically, simvastatin inhibits RhoA and promotes the translocation of HIF-1α to the nucleus of endothelial cells (54).

The regulation of HIF-1α-dependent pathways by lipoproteins has recently been evaluated in cellular models (52). In human pancreatic adenocarcinoma cells, lipoprotein depletion in the cell culture medium leads to the activation of the HIF-1α pathways, but the addition of LDLs, the main cholesterol transporters in the plasma, inhibits the accumulation of HIF-1α by regulating the activity of PHDs enzymes (52). Interestingly, it seems that cholesterol is not directly involved. Rather, it appears that fatty acids released by the lysosomal hydrolysis of cholesterol esters *via* the lysosomal acid lipase decrease the production of mitochondrial ROS, leading to activation of PHDs (52). Conversely, direct addition of free cholesterol (not handled by lipoproteins) in the culture medium of hepatocytes induces HIF-1α through a mechanism involving mitochondrial dysfunction, generation of mitochondrial ROS and NO production (55).

### Regulation of HIF-1α by oxLDLs and Oxysterols

Several independent studies show that macrophage exposure to oxidized lipids markedly induces HIF-1α-dependent pathways (12, 13). Moreover, HIF-1α seems to be required for many of the effects induced by oxLDLs on macrophages including angiogenesis and metabolic reprogramming (13, 56).

Treatment of macrophages with oxLDLs induces the expression of the glucose transporter 1 (GLUT1) and glucose uptake. This effect is mediated by the generation of ROS by the NADPH oxidase NOX2 leading to the activation of HIF-1α (13). OxLDL treatment strongly induces HIF-1α and VEGF in primary human macrophages and promotes tube formation in a co-culture model with endothelial cells (56). HIF-1α also appears to be required for the survival of macrophages exposed to oxLDLs by inducing several anti-apoptotic pathways (57). Beside oxLDL treatment, phagocytosis of cellular debris also activates HIF-1α. Incubation of macrophages with homogenates from human carotid plaques, which contain oxidized cholesterol derivatives, results in a global activation of HIF-1α signaling, including IL-1β production, activation of glycolysis pathways and VEGF production (11, 12).

From a mechanistic point of view, it appears that there are multiple levels of regulation. OxLDLs exert pro-inflammatory effects by activating TLR pathways that are known to promote HIF-1α stabilization (58, 59). The generation of ROS *via* the activation of several NADPH oxidases (NOX2, NOX4) is also involved (60). The different molecular components of oxLDLs have specific effects. OxLDLs as well as the necrotic core of atherosclerotic plaques are rich in oxidized cholesterol derivatives. In particular, 7-oxysterols (such as 7-ketocholesterol) have pro-inflammatory activities and induce the production of ROS by macrophages (61–63). Accordingly, the ability of plaque debris to induce HIF-1α target genes correlates with their oxysterol content (11, 12). Interestingly, this correlation is significant not only for 7-oxysterols but also for other oxysterols such 27-OH and 25-OH cholesterol, which are agonists for the nuclear receptors liver X receptors (LXRs). In line with this observation, an additional mechanism of HIF-1α regulation by oxysterols was recently described. An increase in the expression and in the transcriptional activity of HIF-1α mediated by the activation of LXR was demonstrated (11, 12, 64). Indeed, LXR activation in macrophages by synthetic agonists or with oxysterols-enriched plaque homogenates induce several HIF-1α-dependent pathways, including lipogenesis, IL-1β production, angiogenesis and glycolysis (11, 12, 64). The molecular mechanisms remain to be fully characterized, but it seems that LXR and HIF-1α may directly interact *via* the ligand binding domain of LXR and the oxygen-dependent degradation domain of HIF-1α (64). Accordingly, chromatin immunoprecipitation experiments revealed a co-recruitment of HIF-1α and LXR at the hypoxia response elements (HRE) of target genes (11, 12, 64). Interestingly, this pathway is relevant in the context of atherosclerosis since HIF-1α and LXRα co-localize in the nuclei of macrophages within the plaque (12, 64).

## Impact of Hypoxia and HIF-1α on Cholesterol Homeostasis in Macrophages and Atheroma

**Figure 2:**
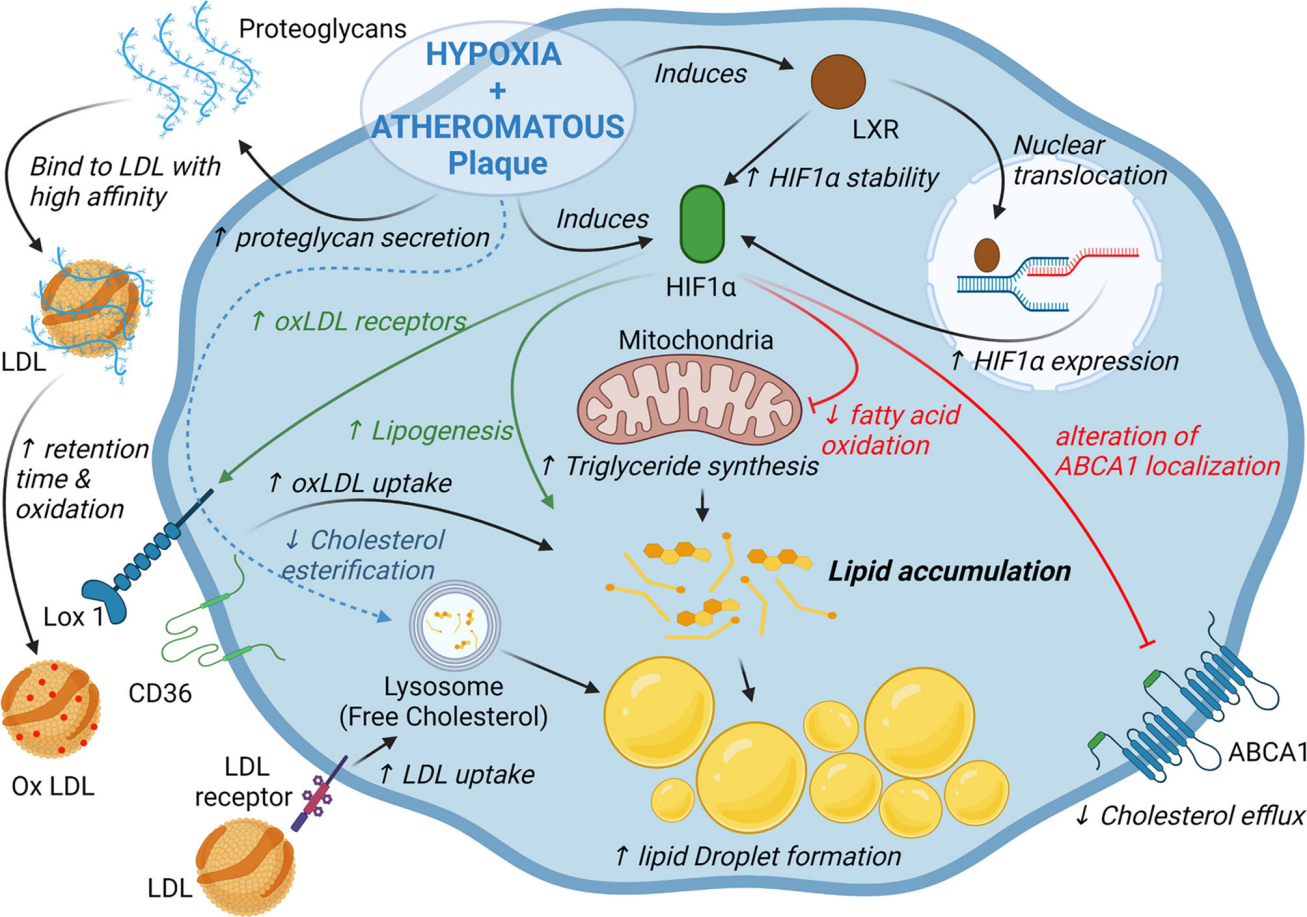
HIF-1α and hypoxia alter lipid homeostasis in macrophages. Activation of HIF-1α dependent pathways promotes triglyceride and cholesterol accumulation in macrophages through activation of lipogenesis, cholesterol synthesis and decrease of fatty acid oxidation. Hypoxia and HIF-1α also promotes low density lipoproteins (LDL) retention as well as oxidized LDL uptake by changing proteoglycan composition and increasing the expression of several scavenger receptors. Finally, in murine macrophages hypoxia/HIF-1α impair cholesterol efflux by altering ABCA1 localization.

As mentioned previously, within the plaque, macrophages can acquire cholesterol and oxysterols through the uptake of oxidized lipoproteins or the phagocytosis/efferocytosis of cells and cellular debris (12, 50, 51). Nevertheless, free cholesterol accumulation is deleterious for the cells because it can form cytotoxic derivatives and pro-inflammatory microcrystals (65). Therefore, free cholesterol is stored in the form of less toxic cholesteryl-esters or is eliminated from the cells by efflux pathways dependent on the ATP Binding Cassette A1 (ABCA1) and ABCG1 transporters (50, 51). Hypoxia and HIF-1α may disrupt the balance between cholesterol input and output, triggering cholesterol accumulation (**Figure 2**). Several studies showed that hypoxic conditions promote the formation of lipid droplets and the transformation of macrophages into foam cells (66, 67). However, the nature of the lipids that accumulate differs according to the studies. For instance, Boström et al. found only an accumulation of triglycerides (TG) without an effect on intracellular cholesterol concentrations in human primary macrophages under hypoxic conditions (66). TG accumulation could be explained by a metabolic reprogramming of macrophages characterized by the activation of lipogenesis along with decrease in fatty acid oxidation (66). Interestingly, this effect could be potentiated by the interaction of HIF-1α with LXR (64). Conversely, Parathath et al. show that hypoxia and HIF-1α increase both TG and sterol concentrations in murine macrophages (10). Whether these discrepancies are related to species differences is intriguing and the subject remains to be further investigated. Interestingly, hypoxia specifically increases the proportion of unesterified cholesterol (10, 67). The underlying mechanisms are not clear, but Acyl-CoA acyl transferase, which is responsible for cholesterol esterification, does not seem to be modulated at the transcriptional level by hypoxia (10, 67). This preferential accumulation of free cholesterol is particularly relevant in the context of atherosclerosis since free cholesterol accumulates in advanced plaques and contributes to inflammation, macrophage apoptosis and necrotic core formation in hypoxic areas in the plaque (65, 68).

While hypoxia and HIF-1α increase the uptake of oxLDLs by macrophages (69), the main scavenger receptors expressed in macrophages are differentially regulated in this context. The scavenger receptor A is inhibited by hypoxic conditions, but hypoxia and HIF-1α induce the expression of the lectin-type oxidized LDL receptor 1 (LOX1) receptor as well as a relocalization of CD36 at the cell surface (69, 70). Accordingly, LOX1 inhibition was shown to decrease oxLDL uptake and foam cell formation under hypoxic conditions (69) (**Figure 2**). Regarding other cell types, hypoxia induces the expression of LDL receptor-related protein 1 and the uptake of aggregated LDL in VSMCs (71) (**Figure 2**). The very low density lipoprotein (VLDL) receptor, expressed in endothelial cells, is also a HIF-1α target and promotes the cellular uptake of LDL and VLDL in hypoxic conditions (72). Finally, hypoxic macrophages secrete proteoglycans that present a higher affinity for LDL which may favor the retention, oxidation and uptake of LDL in hypoxic area in atheromatous plaques (73).

Sterol synthesis is induced in murine macrophages under hypoxic conditions, which is consistent with the induction of HMGCoA Reductase expression, previously described as a HIF-1α target gene (10, 74). Accordingly, statin treatment reduces the accumulation of cholesterol in hypoxic macrophages (10). However, once again the regulation appears to be tissue-specific. In the liver, HIF-1α promotes the degradation of HMGCoA reductase by inducing insulin induced gene 2 (75).

Finally, ABCA1-dependent cholesterol efflux is substantially reduced by hypoxia in a HIF-1α-dependent manner in murine macrophage cell lines and in murine bone marrow-derived macrophages (10). The mechanisms do not appear to be related to a mechanism of transcriptional regulation, since ABCA1 mRNA levels remain unchanged under hypoxic conditions (10). In fact, hypoxia affects the intracellular distribution of ABCA1 which leads to an alteration of ABCA1 localization at the plasma membrane and a reduction of cholesterol efflux (**Figure 2**) (10). By contrast, in primary human macrophages, the HIF-1α/HIF-1β heterodimer was shown to bind to an HRE present in the ABCA1 gene promoter (76). Interestingly, ABCA1 and HIF-1β expression are correlated in macrophages isolated from human atherosclerotic lesions (76).

## Discussion

The studies reviewed here shed lights on the complex interplay between hypoxia, HIF-1α, atherosclerosis and cholesterol homeostasis. Regarding atherosclerosis, the impact of HIF-1α appears to depend on whether we consider the cells present within the plaque or the effects of systemic modulation of HIF-1α (18, 44).

In addition, the mechanisms contributing to the regulation of HIF-1α by cholesterol in macrophages vary according to the considered pathways *i.e*. canonical pathways (cholesterol biosynthesis and LDL uptake) *vs* unregulated cholesterol and cholesterol derivatives uptake in plaque macrophages. These observations further strengthen the interest of identifying the specific mechanisms of HIF-1α activation within the plaque. Additionally, the relative impact of hypoxia *vs* the lipid microenvironment remains controversial. While a wide variety of oxidized lipids and oxysterols are present in atheroma, it remains to be determined which oxysterols and which pathways are more specifically involved. This represents a promising field of investigation and potential targets for new therapeutic strategies.

The impact of HIF-1α on cholesterol metabolism depends on the experimental models. Several studies reveal a marked difference between human and murine macrophages in the accumulation of cholesterol and also in the modulation of cholesterol efflux pathways (10, 66, 76). Similarly, the interplay between HIF-1α and LXRα leading to the activation of glycolysis or to the induction of IL-1β seems to be a feature of human macrophages (12). The understanding of the mechanisms underlying these inter-species differences is still very incomplete.

In conclusion, the relationship between HIF-1α and cholesterol is close, complex, and only partially understood. Nevertheless, there is undoubtedly a reciprocal regulation between HIF-1α and cholesterol in macrophages in the context of atherosclerosis. On one side, HIF-1α modulates macrophage cholesterol homeostasis and on the other, cholesterol-derivatives affect HIF-1α signaling giving rise to a vicious cycle that contribute to a worsening of the atherosclerotic process. It therefore seems crucial to investigate the specific mechanisms leading to HIF-1α activation in macrophages within atheromatous plaques.

## Author Contributions

Writing – Original Draft, DM, CT and DL. Writing – Review and Editing, DM, CT and DL. All authors contributed to the article and approved the submitted version.

## Funding

This work was supported by a French Government grant managed by the French National Research Agency under the program “Investissements d’Avenir” with reference ANR-11-LABX-0021 (LipSTIC Labex).

## Conflict of Interest

The authors declare that the research was conducted in the absence of any commercial or financial relationships that could be construed as a potential conflict of interest.

## Publisher’s Note

All claims expressed in this article are solely those of the authors and do not necessarily represent those of their affiliated organizations, or those of the publisher, the editors and the reviewers. Any product that may be evaluated in this article, or claim that may be made by its manufacturer, is not guaranteed or endorsed by the publisher.
